# An association of metabolic syndrome constellation with cellular membrane caveolae

**DOI:** 10.3402/pba.v4.23866

**Published:** 2014-02-12

**Authors:** Wei-zheng Zhang

**Affiliations:** CMP Laboratory, Port Melbourne, Melbourne, Victoria, Australia

**Keywords:** metabolic syndrome, caveolae, dyslipidemia, hypertension, hyperglycemia, caveolins

## Abstract

Metabolic syndrome (MetS) is a cluster of metabolic abnormalities that can predispose an individual to a greater risk of developing type-2 diabetes and cardiovascular diseases. The cluster includes abdominal obesity, dyslipidemia, hypertension, and hyperglycemia – all of which are risk factors to public health. While searching for a link among the aforementioned malaises, clues have been focused on the cell membrane domain caveolae, wherein the MetS-associated active molecules are colocalized and interacted with to carry out designated biological activities. Caveola disarray could induce all of those individual metabolic abnormalities to be present in animal models and humans, providing a new target for therapeutic strategy in the management of MetS.

Metabolic syndrome (MetS) is defined as a cluster of metabolic abnormalities which is predictive of a high risk of type-2 diabetes and cardiovascular disease (1, 2). Despite slight differences in its definition, the major clinical features encountered are insulin resistance (IR), hyperglycemia, obesity or abdominal obesity, and hypertension and dyslipidemia (3). Although there have been many proposals on the pathogenesis and pathophysiology, any possible mechanism to connect all such abnormalities at cellular and molecular levels has not been delineated. Gathering from available literature, it becomes plausible that the cell membrane domain, caveolae, plays a vital role in the formation of MetS.

The cell bilayer membrane serves as a barrier to the surroundings. Caveola is a special type of lipid raft, which appears as a minute invagination (50–100 nanometer) on the membrane in many vertebrate cell types, especially in endothelial cells (EC) and adipocytes (4–6). These flask-shaped structures are rich in proteins and lipids, and selectively perform an important role in the exchange of information and materials with the environment by acting as coordinators of signal transduction and endocytosis. It has been well documented that caveolae act as signaling platforms, function as a gathering point for numerous signaling molecules, manage cell volume and volume-sensitive signaling in an adipocyte system, act as mechanosensors in many cell types, and control fluctuation through many well-defined signaling cascades (7–9). Some pathogens can enter the target cells via caveolae to preclude a self-degradation in lysosomes (8, 9). The formation of caveolar endocytic vesicles is dependent on the ability of caveolin oligomerization, and sufficient levels of cholesterol and caveolin insertion in plasma membrane (10). Many active transmembrane proteins, including transporters, receptors, enzymes and caveolins, are colocalized within caveolae. They have a distinctive constitution with highly enriched cholesterol, sphingolipids, and 1-, 2-, and 3-caveolins as coating proteins (11). Munday and colleagues have demonstrated a caveolae involved in multi-step trafficking pathway of endocytosis (9), whereby caveolin-containing bodies ‘cavicles’ are transported from the ‘caveosome’ to the cell surface caveolae via microtubules (8, 9).

A new general function of caveola has been revealed by its role in the uptake of different metabolites of lipid and glucose metabolism (12). Caveolae, therefore, come into sight as important centers for many facets of metabolism serving as gateways for the uptake of nutrients across the cell membrane, and as platforms for the metabolic conversion of nutrients, especially in adipocytes and ECs (12). Caveolins, the integral plasma membrane proteins present in caveolae with proven scaffolding, transport, and signaling capabilities, have emerged as key players in lipid dynamics and membrane microdomain disorders apart from its role in the obesity and IR development (13). Currently available literature on caveola and its functions may allude to our understanding of obesity, diabetes, and other metabolic disorders. However, so far there are no definitive published studies on the linkage of MetS and caveolae to raise attention to a mechanism of MetS formation, which have great potential for MetS therapeutic management.

Herein, the aim of this opinion review is to provide an overview from available literature on the participation of caveolae and caveolins as common novel modulators in pathogenesis of the MetS cluster.

The active molecules involved in the MetS cluster are associated with membrane caveolae.

## Insulin resistance

Membrane caveolae play a vital role in compartmentalization of insulin signaling (14). A primary basis of type-2 diabetes is IR caused by a dysfunction of insulin signaling in target tissues. Under the pathological condition of IR, the body produces insulin but cells fail to respond to the normal actions of the insulin hormone due to any efficiency changes of insulin surface receptors. The insulin receptor and part of the downstream signaling mediators can be recruited to and gather at caveolae (15). As part of the signaling, the auto-phosphorylated insulin receptor in primary adipocytes promptly engages with tyrosine phosphorylation of caveolin-1 upon being internalized through a caveolae-mediated process (16), and the process requires membrane cholesterol (17). Lack of caveolae induces IR in animals and human beings (15). The efficacy of insulin signaling in the adipocyte can be firmly relied on the localization of at least two insulin-responsive elements (insulin receptor and GLUT4) to caveolae as well as on a direct functional interaction between insulin receptor and caveolin-1 (18). Approximately 19% of insulin receptor molecules were detected in caveolar regions of adipocytes, and an aberrant ganglioside distribution in the caveolae constitution resulted in IR (19). Disarrays in caveolae lipid composition have been shown *in vitro* to extricate proteins from caveolae, thereby altering their normal functionality and the consequent downstream signaling (20). Furthermore, a high-cholesterol diet can induce an enhanced insulin-induced insulin receptor activation, impair the downstream molecules IRS-1 and Akt activities, and abolish an induction of caveolin-1 tyrosine phosphorylation under the insulin stimulation (20). The atypical interaction between insulin receptor and gangliosides within caveolae can also be one of the molecular pathogeneses of type-2 diabetes (21).

Given that cell volume regulation is significantly adjusted upon adipocyte differentiation which is associated with the number of caveolae present on the cell surface, it is insinuated that caveolae are involved in the pathogenetic mechanism of IR and MetS (22). Although the responses to insulin may vary among cell types, caveolae engage in the process. In RLE-6TN cells, insulin is taken up through endocytosis accompanied by the insulin receptor within caveolae (23). Concomitantly, insulin uptake in EC requires expression of caveolin-1, the main coating protein of caveolae, supporting the motion of caveolae mediating insulin uptake (24). In the adipose cell, caveolin-1 and caveolae also regulate insulin action. Loss function of caveolin-1 reduces maximal insulin response through a lowered stability and diminished expression of the insulin receptor and GLUT4 (25). In the liver cells, caveolin greatly enhances the insulin receptor signaling upon being over-expressed *in vivo* (20), acts as an important regulator of glucose metabolism that can augment insulin signals in the obese mouse livers attributing mostly to an increased insulin receptor activity and the caveolin-mediated direct inhibition of protein tyrosine phosphatase 1B (26). Caveolae and caveolin-1 also play a role in insulin-like growth factor-I receptor internalization and function modulation, respectively (27). A high-cholesterol diet alters caveolin-1 expression *in vivo*, and insulin receptor localization as well as activity (20), which may imply a linkage of hyperlipidemia and IR via caveolae. Strong evidence authenticates that caveolae participate in the pathological origin of IR.

## Hyperglycemia

The major glucose transporter GLUT4 manipulates the blood glucose level and its cellular localization within caveolae is strictly a prerequisite for the functionality of cellular glucose transportation and maintenance (28). Other glucose transporters, GLUT1 and GLUT3 together with hexokinase (a glycolytic and glycogenic priming enzyme) are also found in caveolae (29). In skeletal muscle and adipose tissue, GLUT4 is translocated, upon insulin stimulation, from intracellular storage compartments to cellular membrane within caveolae to trigger the insulin-stimulated glucose uptake, and the process of GLUT4 protein cellular trafficking is precisely regulated by the insulin receptor signals through a series of highly organized membrane trafficking events (30–32). Elevated glucose concentrations diminish the number and the size of caveolae (33), and cause disarray of caveolae constitution (34). In the diabetic lung, however, the EC displays an increased number of caveolae, an enlarged surface area, escalated cholesterol content, and an over-expression (gene and protein) of caveolin-1 (35). Certainly a compensational mechanism needs to be ascertained. The study on the caveolin-1 knockdown adipocytes demonstrated reductions in the insulin-triggered GLUT4 recruitment to the cell surface, in the insulin-stimulated glucose transport, insulin receptor activation caused by a reduced stability, and in expressions of insulin receptors and GLUT4 (25), reversely confirming the roles of caveolae in glucose transportation and metabolism. Further *in vitro* studies also demonstrated that a depletion of cholesterol contained in caveolae can partially inhibit GLUT4 internalization in L6 myoblasts (36), and that IR can be bypassed by manipulating GLUT4 endocytosis for maintaining sufficient GLUT4 on the surface (37). Caveolae formation requires cavin. The cavin-knockout mice are viable and of normal weight but illustrate a significant glucose intolerance, hyperglycemia, and hyperinsulinemia (18). Concisely, glucose transportation and metabolism require its major transporter GLUT4 to exist on the cell surface within caveolae. Any disruption of caveolae could result in hyperglycemia.

## Obesity and dyslipidemia

Obesity, especially visceral obesity, causes IR and is associated with dyslipidemia, impaired glucose metabolism, and hypertension (38). Dyslipidemia commonly accompanies obesity and MetS (39). Caveolae physically involve in fatty acid (FA; 40, 41), triacylglycerol (42), and cholesterol (43) uptakes, in the protection of adipocyte from the lipotoxic effects of elevated FAs, and in the cholesterol synthesis (44). Caveolae within adipocyte are regulated by energy homeostasis. At a state of positive energy balance, expressions of some proteins are accordingly increased to meet the requirements of caveolae structure remoulding coping with the adipose tissue expansion for maximizing the capacity of cellular lipid storage (45). Rising circulating FA levels can also induce a substantial upregulation of caveolar proteins (45). In the obese rats, however, caveolae are not evenly transformed. In the endothelium of arteries, the number of caveolae is declined, whereas, at the ends of smooth muscle cells, caveolae density is intensified (46), which may be aligned with the pathophysiology of obesity on vascular function and carbohydrate metabolism. It also has been demonstrated that lacking functional caveolae can cause dyslipidemia and can result in reduced fat storage accompanied by smaller sizes of the fat cells in mice and humans (45). The cellular FA utilization and the initial metabolic regulation require a precise control on the cellular FAs uptake via membrane. In many tissues, caveola facilitates a major fraction of FA uptake (47) and conversely higher levels of FAs can induce an enhanced density of caveolae in obese rats (48). A study on dietary intake of saturated fat implies that the saturated fats can intensify the levels of sphingolipids in cardiac cell membranes resulting in a disruption of the caveolae composition and can significantly reduce the systolic contractile performance as well as caveolin-1 contents (49). Investigation on cavin-knockout mice, which has a disrupted caveolae composition, demonstrated an extensively shrunken adipose tissue mass (18). The membrane lipid environment affects caveolin isoforms (49). In the arterioles of obese rats, only caveolin-1 and -2 oligomer expressions, but not their monomers and caveolin-3, are elevated (46). Sphingomyelin is one of the major phospholipids of caveolae. Conformational changes in plasma membrane sphingomyelin can induce obesity and type-2 diabetes (50). In the caveolin-1 null mice, triglyceride and free FA levels are significantly elevated (51), and a modestly increased rate of lipolysis together with a diminished cellular integrity are evidenced (45). In contrast, caveolin-1-knockout mice exhibit a lean phenotype with an overt resistance to the diet-induced obesity, whereas, caveolin-3-knockout mice show a marked IR accompanied by an increased body weight and adiposity in a normal diet (13).

Cluster of Differentiation 36 (CD36), a member of the class B scavenger receptor and FA transporter family within the caveolae domain on cell surface, binds many ligands including oxidized low-density lipoprotein (OxLDL) (52), native lipoproteins (53), oxidized phospholipids (54), and long-chain FAs (55). Together with Caveolin-1, CD36 participates in adipocyte FA uptake and metabolism, and both are coordinately involved in lipid droplet formation (50) and associated with long-chain FA uptake (56). CD36 membrane levels and the turnover are abnormal in diabetes, causing a dysfunctional FA utilization. In addition, polymorphism of the CD36 gene has been shown to influence its susceptibility to MetS (47). OxLDL impacts on membrane rafts and caveolae in the distribution of different membrane raft constitutes, on membrane cholesterol composition, and on lipid packing of different membrane domains (57). OxLDL moves CD36 from low to high buoyant density membrane fractions together with caveolin-1 (58). Upon uptake OxLDL, CD36 can segregate them at the cell surface, interfering with intracellular trafficking and degradation (59). Conversely, Caveolin-1 plays an important role in recruiting fatty acid translocase (FAT)/CD36 to caveolae, and in regulating FA uptake via cellular surface availability of FAT/CD36 (60). Caveolae integrity has been shown to directly affect the CD36 function and blood pressure regulation (60). Both high-density lipoprotein cholesterol (HDL) and low-density lipoprotein cholesterol (LDL) participate in maintaining the lipid environment in caveolae (61, 62). The former upholds the caveolae integrity, while the latter, especially oxLDL, acts on an opposed effect of inducing membrane caveolae internalization (62).

A large amount of evidence suggests that the abnormality of lipids could affect caveolae composition and functionality, which would further disturb FA metabolism in the development of dyslipidemia and obesity.

## Hypertension

Although many abnormalities are suggested to be the pathogenesis of hypertension, endothelial nitric oxide synthase (eNOS) existing within cell membrane caveolae, which produces nitric oxide (NO), is well defined as a major factor in maintaining vascular function. A reduced production of NO can cause hypertension (62–64). An aberrant regulation on eNOS together with an associated NO/SNO release is directly linked with various vascular diseases. eNOS is primarily localized on the Golgi apparatus and plasma membrane caveolae in EC (64). It functions to produce NO only when it presents on the cell surface within caveolae (62). Caveolae transduce eNOS/NO/SNO cardioprotective signaling in the heart (65). The scaffolding domain of caveolin-1 serves as an endogenous negative regulator of eNOS function (66), which is supported by the evidence of that the caveolin-deficient animals show an unopposed NO production promoting vessel dilation (67). In addition, reactive-oxygen-species-dependent eNOS activation and eNOS uncoupling are critically regulated by caveolin-1 (67). Alpha-galactosidase A participates in glycolipid metabolism and caveolin assembly. Research on alpha-galactosidase A-knockout mice has demonstrated that the animals had high molecular weight caveolin oligomers reduced and detached caveolae in the ECs (68).

Caveolae also associate functionally and anatomically with many other active molecules of blood pressure ‘regulators’. Specifically, caveolae act as binding sites for calcium ions (as a major Ca(2+), influx is through L-type calcium channels within caveolae signaling domains) (69), participate in regulating both pumping and signal transducing functions of Na(+)/K(+)-ATPase (70), are involved in AT1receptor internalization (71), influence calcium-activated chloride channel properties (72), and regulate alpha1-adrenergic receptor signaling (73). In addition, caveolae engage critically in endothelial signal transduction from shear stress to vasodilator production and release (74). The endothelium-dependent shear-stress-mediated vasodilation requires the integrity of caveolae. In the Cav-1^−/ −^ endothelium, the shear-stress-mediated vasodilators including NO, epoxyeicosatrienoic acids, and prostaglandins are deficient (74). In view of that, caveolae and caveolin participate in the regulation of blood pressure. Deformity of caveolae can cause hypertension.

## Some other caveolae associated active molecules involved in MetS

Many other active molecules within caveolae have also been revealed to be associated with MetS. Vitamin D (25(OH)D) deficiency has been demonstrated to be associated with a high risk of MetS involving in a higher serum triglyceride, elevated fasting glucose, and induced IR (75). Vitamin D initiates rapid cellular responses through a putative plasma membrane-associated vitamin D receptor (VDR), which is sited on caveolae (Pdia3) (76). VDR regulates gene expression of the encoding proteins that propagate the traditional genomic functions of vitamin D, and the quick response of vitamin D binding with VDR delays chronic diseases of aging such as cancer, vascular disease, type-1 and -2 diabetes, arteriosclerosis, osteoporosis, and infection (77). Binding with VDR at different cellular locations can selectively mediate both genomic and cellular responses (78). VDR can be recruited on cell surface within caveolae upon stimulation of vitamin D (79). Elevated 7-dehydrocholesterol (a cholesterol precursor which is converted to vitamin D3 in the skin) in the caveolar membrane can induce a defective caveolar signaling (80). As its roles in metabolism become evident, a larger vitamin D clinical trial has been recently approved by NIH on its prevention of diabetics (news release: http://www.nih.gov/news/health/oct2013/niddk-21.htm), so the final results of any promises of therapeutic benefits beyond healthy bones are pending.

The beneficial metabolic actions of estrogen-based therapies are mainly mediated by estrogen receptor (ER). ER has been suggested to be involved in obesity (81). Upon binding with ER at caveolae, estrogen can induce NOS activation (61) and play a role in the maintenances of body metabolism (82). The ER knockout-mouse electively developed an accelerated weight gain, massive adiposity, severe IR, and glucose intolerance (83). 17β-estradiol administration regulates some key metabolic genes in insulin-sensitive tissues and confers a strong protection against high-fat diet-induced metabolic disturbances (83). Despite the beneficial estrogenic effects in reversing some of the MetS symptoms, an increasing body of evidence now links estrogenic signaling with MetS (84). Targeted estrogen delivery on ER reverses MetS (85). Specifically, loss of ER signaling leads to IR and obesity in animals (86).

Inflammation can also be involved in caveolae impinging on MetS. It has been suggested that NF-*κ*B activation may participate in chronic inflammation, IR, endothelial dysfunction, hypertension, and dyslipidemia (87). NF-*κ*B activation, however, is associated with caveolae and specifically requires caveolin-1 (88). Moreover, during the inflammation process and under hyperglycemia, the apoptosis of macrophages might occur, leading to the spreading of lipids of cell membrane caveolae from macrophages into intracellular spaces in the vessel wall (33). The damaging vascular wall can insert detrimental effects on the development of all relevant abnormalities of MetS. Furthermore, exercise could reduce MetS and exert anti-inflammatory effects (89), which may act through increasing shear-stress to regulate caveolae signal transduction (74).

## Future studies and prospective applications

As the joint AHA-NHLBI statement has recommended MetS as a clinical entity, and for its involvement in all MetS relevant abnormalities (90), caveolae are a potential therapeutic target for MetS. Controlling obesity by reducing hyperlipidemia and LDH (especially oxLDL) level and increasing physical activities, drugs for reducing blood lipids and/or blood pressure have shown benefits in the management of MetS. At the molecular level, manipulating caveolins/caveolae can affect NO production, insulin action, and lipid and hormone metabolisms. Therefore, caveolae together with its components may become useful targets for treating MetS. For example, L-Arginine supplementation could induce its transporter, cationic amino acid transporter 1, recruited into caveolae and concurrently trigger caveolae translocation onto cell surface (62) with subsequent effects on insulin sensitivity and reduce body fat content (91). Preliminary studies in our laboratory on caveolar cellular internalization have demonstrated a potential regulation on the function of caveolae in EC after treatment with nutrients and chemicals (unpublished data). Extensive research on the regulation of caveolae function, especially on cellular trafficking both *in vivo* and *in vitro*, is warranted. In conclusion, the rising prevalence of the MetS especially in youth (92) and its associated abnormalities is a major public health problem. However, the physiopathology of MetS remains to be ascertained. Information from published studies suggests that all MetS-associated metabolic abnormalities have a common pathogenesis origin emanated from cell membrane caveolae, which presents a new therapeutic strategy for MetS.
